# Early Referral to Nephrological Care and the Uptake of Peritoneal Dialysis. An Analysis of German Claims Data

**DOI:** 10.3390/ijerph18168359

**Published:** 2021-08-07

**Authors:** Isabell Schellartz, Sunita Mettang, Arim Shukri, Nadine Scholten, Holger Pfaff, Thomas Mettang

**Affiliations:** 1 Institute of Health Care Research, Rhineland State Council, LVR-IVF, 51109 Cologne, Germany; 2 Faculty of Human Sciences and Faculty of Medicine, Institute of Medical Sociology Health Services Research and Rehabilitation Science (IMVR), University of Cologne, 50933 Cologne, Germany; nadine.scholten@uk-koeln.de (N.S.); holger.pfaff@uk-koeln.de (H.P.); 3 Betriebskrankenkasse (BKK) Linde, 65187 Wiesbaden, Germany; sunita.mettang@gmx.de; 4 Institute for Health Economics and Clinical Epidemiology (IGKE), University of Cologne, 50935 Cologne, Germany; arim.shukri@uk-koeln.de; 5 Kidney Center Wiesbaden, 65191 Wiesbaden, Germany; thomas.mettang@gmx.de

**Keywords:** early referral, hemodialysis, peritoneal dialysis, treatment choice, pre-dialysis care, claims data, statutory health insurance

## Abstract

Background: Hemodialysis (HD) and peritoneal dialysis (PD) are medically equivalent alternatives for symptomatic therapy of end-stage renal disease (ESRD). An early referral (ER) of patients with chronic kidney disease (CKD) to a nephrological specialist is associated with a higher proportion of patients choosing PD. Germany historically shows a low PD uptake. This article is the first investigation into the impact of ER on the uptake of PD, using a large German claims database. Methods: Claims data of 4727 incident dialysis patients in 2015 and 2016 were analyzed. Accounting codes for nephrological care and dialysis modalities were identified. Their first documentation was defined as their first encounter with a nephrologist and their first dialysis treatment (HD or PD). ER was determined as receiving nephrological care at least six months before the first dialysis. A multivariate logistic regression model with adjusted odds ratios (AOR) investigates the impact of ER, outpatient dialysis start, age, comorbidities, and sex on the chance for PD. Results: Forty-three percent were referred to the nephrologist six months before their first dialysis (ER). Single tests, as well as the adjusted multivariate logistic regression, highlighted that ER significantly increases the chance for PD. In the multivariate model, the uptake of PD was associated with ER (AOR = 3.05; *p* < 0.001; 95% CI = 2.16–4.32), outpatient dialysis start (AOR = 0.71; *p* = 0.044; 95% CI = 0.51–0.99), younger age (AOR = 0.96; *p* < 0.001; 95% CI = 0.95–0.97), and fewer comorbidities (AOR = 0.85; *p* < 0.001; 95% CI = 0.44–1.58). Conclusions: ER of patients with CKD to a nephrologist increases PD uptake. It gives both nephrologists and patients enough time for patient education about different treatment options and can contribute to informed decisions about the dialysis treatment.

## 1. Introduction

Patients with chronic kidney disease (CKD) suffer from a series of clinical problems. Their bodies are not sufficiently detoxified. When patients with CKD reach a certain point in the progression of their disease (end-stage renal disease, ESRD), they face a choice among different renal replacement therapies (RRTs). The two most commonly used types of dialysis are hemodialysis (HD) and peritoneal dialysis (PD).

With HD, toxins in the blood are filtered by a machine. It is usually conducted in ambulatory dialysis centers three times a week, each session taking four hours. PD can be applied by the patient themselves at home or in any clean environment. In order to enable the detoxication, a dialysate is filled inside the peritoneum and must be replaced regularly. This procedure must usually be conducted four times a day and takes about 30 minutes. Between the dialysate replacements, the dialysate stays in the body and enables detoxication. As the treatment can be arranged flexibly in terms of time and place, PD can enable a more autonomous life [1]. Both dialysis treatments are symptomatic but life-sustaining. HD and PD are considered medically equivalent in terms of survival [2,3].

The progression of CKD and the time in which the need for dialysis becomes apparent can vary among patients. ESRD is usually reached with a glomerular filtration rate (GFR) < 15 [4]. The lower the GFR, the lower the level of kidney function is. The aim is to provide patient education, information about different RRT options, and dietary counseling, as well as psychological and social care. Therefore, an early referral (ER) of patients to a nephrological specialist at least one year before the expected dialysis starts, and with a GFR ≤ 30 mL/min, is recommended [4]. Early care by a nephrological specialist can positively influence the progression of CKD [5]. ER is also associated with better clinical outcomes such as a lower risk for mortality [6,7,8,9,10], better quality of life [11,12], and a lower hospitalization rate [8]. CKD patients who were referred late (late referral, LR) were more likely to suffer from worse psychosocial adjustment [4]. LR to a nephrological specialist is often seen as a barrier to PD [13]. The literature shows that CKD patients with ER to nephrological care are more likely to conduct PD [11,14,15,16,17,18]. A pooled analysis of a systematic Cochrane review reported patients with ER were twice as likely to choose PD [8]. In these studies, ER was operationalized as care by a nephrological specialist between one month and one year before the patient’s first dialysis.

Germany historically shows a low proportion of patients with ESRD dialyzing via PD of 6.7% [19]. The PD ratio generally depends on patient- and physician-related factors as well as the socioeconomic context of the healthcare system [20,21,22,23]. Among others, this includes whether PD or the transport to in-center HD are covered by health insurance providers. The compulsory statutory health insurance (SHI) in Germany reimburses all dialysis options, covers the transport to in-center HD, and providers also support a higher utilization of PD [24]. Hence, framework conditions seem to be in place, and researchers have been trying to grasp, for years, why these reasons are strong drivers, especially in Germany.

Therefore, the aim of the current study is to investigate the association between ER and PD uptake with a large, unselected population of those insured by SHI in Germany. The underlying research question was: Does the ER of patients with CKD to a nephrological specialist increase the uptake of PD? To our knowledge, this article presents the first analysis on this topic using a large claims database reflecting the patient flow with all physician encounters and treatments in the healthcare sector. Thus, our study adds deeper insight into the referral situation in Germany from a system perspective, as well as its impact on PD uptake.

## 2. Materials and Methods

Ninety percent of the German population is insured with the compulsory SHI [25]. Each SHI fund is obliged to contract every person [26]. Therefore, their claims data offers an unselected view on the uptake of services of a broad collective of that population. German SHI claims data have also been used for investigations in nephrology [27,28,29] and when dealing with patients with other chronic conditions [30,31]. We analyzed anonymous claims data of two of the largest SHIs in Germany: DAK-Gesundheit (Deutsche Angestellten Krankenkasse) and SBK (Siemens Betriebskrankenkasse). These funds cover 5.6 and 1.1 million insured members.

The database for this study consists of all medical encounters of 34,200 patients with ESRD from 2012 to 2016, i.e., those who had at least one documentation of International Classification of Diseases (ICD) code N18.5 between 2012 and 2016. The investigation was part of the MAU-PD study (Multidimensional analysis of causes for the low prevalence of ambulatory peritoneal dialysis in Germany) [32]. The study was funded by the Federal Joint Committee’s innovation fund (Funding No 01VSF16036). It was ethically approved by the ethical committee of the University Hospital of Cologne, which considered all aspects of the Declaration of Helsinki.

### 2.1. Structure of German Claims Data

In Germany, the reimbursement of outpatient medical services covered by the SHI is based on a codebook of different medical services (Einheitlicher Bewertungsmaßstab, EBM) [24]. In this codebook, each EBM code corresponds to a certain medical service. For inpatient admissions, each OPS code (Operationen- und Prozedurenschlüssel) corresponds to a certain medical procedure, e.g., the conduction of a dialysis treatment [33]. The transfer of EBM and OPS codes to the SHI is relevant for the reimbursement of a service. All medically necessary services are covered by the SHI and are therefore contained in the claims data with OPS and EBM codes. These reimbursement codes make it possible to use claims data to analyze the care provided to patients and its effects.

### 2.2. Identification of the First Dialysis and the Different Dialysis Treatments

We selected the OPS and EBM codes relevant for the reimbursement of dialysis (Supplementary Table S1) [24]. In our case control study, HD and PD patients were retrospectively identified depending on whether the code of their first dialysis was an in-center HD or home-based PD. With additional reimbursement codes for special dialysis forms, we excluded patients conducting special types of dialysis: intermittent peritoneal dialysis (IPD) and home hemodialysis (HHD). IPD is conducted in special cases and also at the dialysis center. HHD patients usually start with in-center HD and later switch to HHD. We cannot directly distinguish between the pre-dialysis care and the time between the first HD and the implementation of HHD as a direct preparation time for HHD. Therefore, reimbursement codes in SHI claims data are not adequate to assess the impact of ER on the uptake of HHD. Hence, the difference between in-center HD and home-based PD is of interest for the claims data analysis of referral.

### 2.3. Identification of the First Encounter with a Nephrologist

The identification of outpatient specialist care by a nephrologist before the first dialysis is based on the documentation of the continuous care of a CKD patient with a GFR below 40 mL/min (Supplementary Table S1) [24]. Only nephrologists are authorized to bill the continuous care of a CKD patient. Further, the nephrologist is obliged to educate the patient about dialysis and/or transplantation. In our analysis, the first documentation was determined as the first encounter of a CKD patient with a nephrologist. This scheduled the time of referral and the beginning of the care by a nephrological specialist. Patients who did not have any documentation in their data since 2012 were treated as having no encounter with a nephrologist before their first dialysis. There is a different reimbursement code for the continuous care of patients who have had a kidney transplant. Hence, our approach excluded patients who have had a kidney transplant.

### 2.4. Personal Characteristics

Year of birth and sex were also provided in the claims data. We calculated the patient’s age at their first dialysis with their year of birth and the date of the first dialysis. The non-age-adjusted Charlson Comorbidity Index (CCI) was calculated for the time of each patient’s first dialysis. The CCI weighs 17 comorbid conditions, e.g., diabetes or congestive heart failure, to assess the risk for mortality [34]. Higher scores indicate a higher mortality risk.

### 2.5. Analysis

We included incident HD and PD patients in 2015 and 2016 in our study population. Our case-control study retrospectively focuses on the referral to the nephrologist and, therefore, on the time before the first dialysis. This gave us the possibility to analyze the medical encounters since 2012. The time between a patient’s first dialysis and their first encounter with a nephrologist was calculated.

In the literature, ER was operationalized as care by a nephrological specialist between one month and one year before a patient’s first dialysis [8,11,14,15,16,17,18]. Hence, patients who had their first encounter with a nephrologist within six months (≤180 days) before their first dialysis were classified as LR, and those whose first encounter exceeded six months were classified as ER. Using the clinical binary classification allowed us to correctly assign those patients who already received nephrological care before 2012 to the ER group.

The aim is to investigate the impact of the category of referral on the choice between HD and PD. Chi-square tests and Wilcoxon–Mann–Whitney tests were applied to investigate significant differences between the category of referral (ER or LR), type of dialysis (HD or PD), sex, age, setting (outpatient or inpatient), and CCI, respectively. Patients starting their dialysis in an inpatient setting often suffer from a worse medical condition. Adjusted odds ratios (AORs) for the chance for PD (outcome: HD or PD) were calculated in a multivariate logistic regression model. To adjust for possible confounders, the independent variables besides the category of referral (ER or LR) were sex, age at first dialysis, setting (outpatient or inpatient), and the CCI. The significance level for all tests was 0.05. The 95% confidence intervals (CI) and R^2^ were reported. Data analysis was conducted using Stata 16 (StataCorp., College Station, TX, USA) [35].

## 3. Results

**Figure 1:**
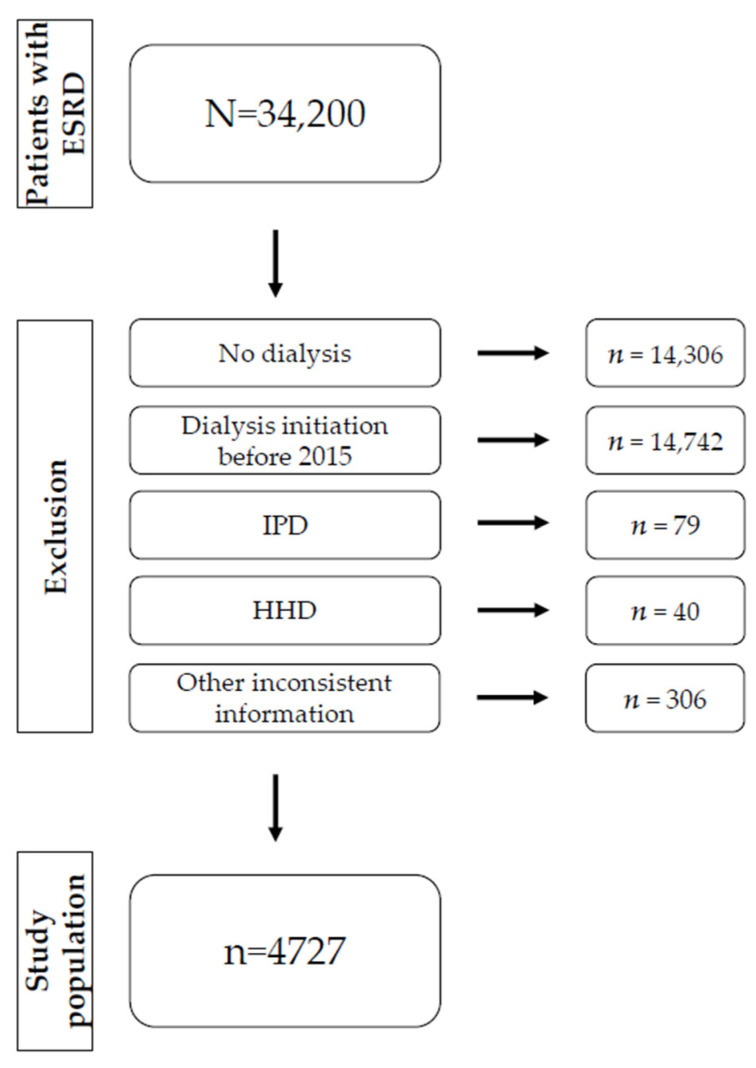
A flow chart about the exclusion of participants. Notes: Patients with ESRD of the SHIs had at least one documentation of the ICD code N18.5 between 2012 and 2016. ESRD patients who did not start a dialysis treatment, started dialysis before 2015, conducted IPD or HHD, or had any inconsistent information were excluded (see method section).

After excluding patients who did not meet inclusion criteria (Figure 1), the study population consisted of 4727 patients. In 2015, 2326 patients started with dialysis treatment, and in 2016, 2401 patients began treatment. Twelve percent were contracted to SBK and 88% to DAK-Gesundheit. Forty-two percent were female. The mean age at first dialysis was 71 years (median 75). The youngest patient was 19, and the oldest was 97. Twenty-nine percent of the dialysis initiations were performed in an outpatient setting. In 96.3% of the cases, patients started with HD, and the remaining 3.7% began with PD. Mean CCI was 8.0. Forty-three percent had their first encounter with a nephrologist more than six months before their first dialysis and were thus categorized as patients with ER. A total of 2673 patients (57%) have been referred within six months before their first dialysis (LR).

Single test results of differences between HD and PD as well as ER and LR are displayed in Table 1 and Table 2. The proportion of outpatient dialysis initiations was higher among PD patients than HD patients (51% vs. 28%, *p* < 0.001). A Wilcoxon–Mann–Whitney test showed PD patients to be significantly younger at their first dialysis than HD patients (60 vs. 72 years, *p* < 0.001). They also had fewer comorbidities (5.5 vs. 8.0, *p* < 0.001). The ratio of PD patients was significantly higher among those with ER than those with LR (5.8% vs. 2.0%, *p* < 0.001). A univariate logistic regression shows that patients referred early were 31% more likely to start their dialysis therapy within an outpatient setting (OR = 0.69, *p* < 0.001, 95% CI 0.27–0.35). Patients having their first dialysis within an inpatient setting were significantly older (mean 72 vs. 69 years, *p* < 0.001). The mean CCI of patients having a first inpatient dialysis was higher (8.4 vs. 6.9, *p* < 0.001).

As HD and PD patients significantly varied in sex, age, setting (inpatient or outpatient), and CCI, these factors were added as confounding variables in a multivariate logistic regression model that examines the influence of ER on PD uptake. Results are displayed in Table 3. The AOR in this model adjusted for sex, age, setting, and CCI highlights that patients with ER had a threefold increased chance for PD (AOR = 3.05; 95% CI 2.16–4.32; *p* < 0.001). Sex was not significant in this model. One additional year of life meant a 4% lower chance for PD (AOR = 0.96; 95% CI 0.95–0.97; *p* < 0.001). An AOR of 0.71 in inpatient admission meant a 29% lower chance for PD (AOR = 0.71; 95% CI 0.51–0.99; *p* = 0.044). Each point higher on the CCI resulted in a 15% lower chance for PD (AOR = 0.85; 95% CI 0.44–1.58; *p* < 0.001). Pseudo R² was 0.135.

## 4. Discussion

We investigated the impact of ER to nephrological treatment on PD uptake with a claims data analysis. Forty-three percent of the patients were referred to the nephrologist more than six months before their first dialysis. These patients were categorized as ER. The adjusted multivariate logistic regression model highlights that ER significantly increases PD uptake. Adjusted for potential confounders, patients with ER had a threefold increased chance for a PD. The confounder’s age, setting (inpatient or outpatient), and CCI also maintained their impact on the chance for PD. In the multivariate regression model, PD uptake was associated with ER, outpatient setting, younger age, and lower CCI.

The operationalization of ER in the literature varied between one month and one year [8,11,14,15,16,17,18]. The different definitions discussed in a review [16] considered the time for medical preparation as a reason for the narrow definition of 3–4 months. However, they also highlighted that guidelines’ referral recommendations and other wider definitions are oriented toward the prevention of early CKD progression. As this investigation focused on the choice between HD and PD, we assumed that a period of more than six months offers the possibility for an informed decision. Our results confirmed recent studies: patients with ER to care by a nephrological specialist have a higher chance for PD. Effect sizes vary among these studies. A pooled analysis reported twice a chance [8]. Our adjusted model from a system perspective revealed ER patients have a three-times increased chance for a PD.

The base for ER operationalization was the documentation of the continuous care of CKD patients with a GFR < 40. The opportunity for CKD patients in this state of progression to receive care by a nephrological specialist even allows for an earlier referral than recommended (GFR < 30) [4]. The results of our study indicate that the framework condition given by the German healthcare system contributes to ER. Forty-three percent of patients with ER and its effect on PD uptake highlighted the relevance of long-term nephrologist care for patients with CKD. Information about different RRT options is fundamental for pre-dialysis care by nephrologists and is thus a mandatory and crucial part of the long-term nephrologist care defined by this code. Six months of continuous nephrological care seems sufficient to educate patients about different RRTs. Nephrologists can provide information about different treatment options and involve relatives. The education gives both patients and nephrologists time to discuss different RRTs and weigh the advantages and disadvantages. Patients may have the opportunity to meet a patient who is already on PD. A patient’s situation at home can be prepared. For a technically planned dialysis start, at least a couple of weeks are required for shunt or catheter implantation. PD also needs a certain “break-in period” between the implantation and the first dialysis. As the goal is to enable a shared decision and not to increase the PD ratio itself, ER is valuable to informed decision-making for an RRT.

The referral to care by a nephrological specialist in an early stage of CKD is not only meaningful with regard to the start of the dialysis treatment. It is also a relevant issue because ER can slow down the progression of CKD, and it offers a way to prevent ESRD and the start of a dialysis treatment [5,16,27]. As this is difficult to assess with claims data, we focused on investigating the association between ER and PD uptake.

Despite the possibility of nephrological treatment and a close care network in Germany, there is still a high proportion of patients with LR (57%). Baer and colleagues summarized reasons for LR [16]. Besides medical reasons, they also stress possible personal factors of patients, such as age and comorbidities. General practitioners could misinterpret the benefits of an RRT for the elderly, which can end in LR. Information campaigns for general practitioners about the comorbidities of chronic diseases and CKD can help to decrease the high proportion of LR [16].

The ambulatory and stationary sectors are strongly separated in Germany. An inpatient dialysis start may be an indicator of an urgent start due to a patient’s acute medical condition during an inpatient admission. In our sample, the proportion of patients starting their dialysis in an inpatient setting is higher than those with LR (82% vs. 57%, respectively). There are data in the literature suggesting that HD and PD are both feasible and safe alternatives for urgent dialysis [36,37,38,39]. Nevertheless, an urgent start of dialysis is often seen as a barrier to PD [13]. The significantly lower PD ratio within inpatient dialysis initiations in our study also seems to indicate that PD is still not generally adopted as an equivalent alternative for an urgent dialysis start.

Our results corroborate the findings of previous studies about the effect of ER on PD uptake based on a large sample from a system perspective. The adjusted model highlights that ER has a major impact on PD uptake. Adjusted odds ratios indicate that ER is more important than a patient’s underlying comorbidities, age, and setting. Despite the close care structure in Germany, there is still a high proportion of patients with LR. Further investigations should pay attention to reasons for the high proportion of LR to reduce this barrier to informed decisions.

### Strengths and Limitations

This study added a system perspective on the ER of patients with CKD by analyzing the claims data of two large German SHIs. About 90% of the German population is contracted with SHIs [25], with 2401 patients starting dialysis only in 2016 [19]. DAK-Gesundheit and SBK cover 6.6 million people [40,41]. The large database allowed us to ensure medical encounters and treatment groups with valid criteria. The PD proportion in our sample was lower than the one stated by the official national report for 2016 (3.7% vs. 6.7%, respectively) [19]. This may be due to the fact that our calculation was based on the number of dialysis patients, whereas the national report counted the number of dialysis treatments. Nevertheless, the large database still enabled us to examine a large sample of 4727 incident dialysis patients in 2015 and 2016.

Due to the compulsory SHI, German SHI claims data provide a representative sample. It is well defined how diseases and treatments are to be coded. Therefore, we expect our results can be confirmed by other SHI funds data and that they have high external validity.

We are aware that our research comes with limitations concerning SHI claims data analysis in general. Such data only consists of procedures relevant to reimbursement. Parts of treatment that are not reimbursed were not included. Consistent with practitioners’ routine coding practice, we used a valid code for the counseling of pre-dialysis CKD patients. This code cannot reflect the extent to which patients have actually been informed. However, even with the selected measurements in primary data, the intensity and time spent on educational counseling over a longer period of time are difficult to measure. The reimbursement code can reflect pre-dialysis nephrological treatment, and this is what the referral category is meant to measure. The precise reimbursement codes made it possible to investigate referrals to nephrologists and pre-dialysis treatments from a system perspective.

The multivariate model’s variables predicted 13.5% of PD uptake. As mentioned before, there are several factors on patient-, physician-, and system-level relevant for the choice between HD and PD [20,21,22,23]. The Pseudo R² in our model confirms that ER is one of them, but not the only factor impacting on PD uptake.

## 5. Conclusions

The considerable number of patients with ER led us to conclude that framework conditions in the German healthcare system support the early care of patients with CKD by nephrologists. The results of the first claims data analysis with a broad collective of those covered by SHIs confirmed the existing evidence: ER increases PD uptake in Germany. ER gives both nephrologists and patients time for patient education about different RRTs and contributes to informed decisions about the dialysis treatment.

## Figures and Tables

**Table 1:** Characteristics of HD and PD patients.

Characteristic	HD	PD	*p*-Value
Female, %	42	43	0.695 *
Age in years, mean ± stand. dev.	72 ± 13.2	60 ± 15.0	0.000 **
Outpatient setting, %	28	51	0.000 *
Patients with ER, %	42	69	0.000 *
CCI, mean	8.0	5.5	0.000 **

* Chi-square test, ** Wilcoxon–Mann–Whitney test, HD: hemodialysis, PD: peritoneal dialysis, stand. dev.: standard deviation, ER: early referral, CCI: Charlson Comorbidity Index.

**Table 2:** Characteristics of patients with ER and LR.

Characteristic	ER	LR	*p*-Value
Female, %	40	43	0.029 *
Age, mean ± stand. dev. (median)	72 ± 13.0 (75)	71 ± 13.8 (74)	0.002 **
Outpatient setting, %	43	18	0.000 *
PD, %	5.8	2.0	0.000 *
CCI	8.0	7.9	0.918 **

* Chi-square test, ** Wilcoxon–Mann–Whitney test, stand. dev.: standard deviation, ER: early referral, LR: late referral, PD: peritoneal dialysis, CCI: Charlson Comorbidity Index.

**Table 3:** Results of the multivariate logistic regression model (outcome: HD or PD).

Independent Variable	Odds Ratio	*p*-Value	95% CI
Cons	0.83	0.572	0.43–1.58
ER (Reference: LR)	3.05	0.000	2.16–4.32
Female (Reference: Male)	1.0	0.812	0.76–1.43
Age	0.96	0.000	0.95–0.97
Inpatient setting (Reference: outpatient setting)	0.71	0.044	0.51–0.99
CCI	0.85	0.000	0.44–1.58

CI: Confidence interval, CCI: Charlson Comorbidity Index, ER: early referral, LR: late referral.

## Data Availability

Restrictions apply to the availability of these data. Data were obtained from DAK-Gesundheit and SBK and are available with the permission of DAK-Gesundheit and SBK.

## Supplementary Materials

The following are available online at https://www.mdpi.com/article/10.3390/ijerph18168359/s1, Table S1. Dialysis-associated EBM and OPS codes and their operationalization for the analysis.

## Trial Registration

The MAU-PD study (Multidimensional analysis of causes for the low prevalence of ambulatory peritoneal dialysis in Germany) is registered at the German Clinical Trials Register. (DRKS-ID: DRKS00012555; Link: https://www.drks.de/drks_web/setLocale_EN.do (accessed on 3 July 2021); Date of Registration in DRKS: 4 January 2018).

## References

[1] Sitjar-Suñer M., Suñer-Soler R., Masià-Plana A., Chirveches-Pérez E., Bertran-Noguer C., Fuentes-Pumarola C. (2020). Quality of Life and Social Support of People on Peritoneal Dialysis: Mixed Methods Research. Int. J. Environ. Res. Public Health.

[2] Wong B., Ravani P., Oliver M.J., Holroyd-Leduc J., Venturato L., Garg A.X., Quinn R.R. (2018). Comparison of Patient Survival Between Hemodialysis and Peritoneal Dialysis Among Patients Eligible for Both Modalities. Am. J. Kidney Dis..

[3] Zhou H., Sim J.J., Bhandari S.K., Shaw S.F., Shi J., Rasgon S.A., Kovesdy C.P., Kalantar-Zadeh K., Kanter M.H., Jacobsen S.J. (2019). Early Mortality Among Peritoneal Dialysis and Hemodialysis Patients Who Transitioned with an Optimal Outpatient Start. Kidney Int. Rep..

[4] Levin A., Stevens P.E., Bilous R.W., Coresh J., de Francisco A.L.M., de Jong P.E., Griffith K.E., Hemmelgarn B.R., Iseki K., Lamb E.J. (2013). Kidney Disease: Improving Global Outcomes CKD Work Group. KDIGO clinical practice guideline for the evaluation and management of chronic kidney disease. Kidney Int. Suppl..

[5] Chen J.-H., Chiu Y.-W., Hwang S.-J., Tsai J.-C., Shi H.-Y., Lin M.-Y. (2019). Effect of nephrology referrals and multidisciplinary care programs on renal replacement and medical costs on patients with advanced chronic kidney disease: A retrospective cohort study. Medicine.

[6] Foley R.N. (2017). Epidemiology and Risk Factors for Early Mortality After Dialysis Initiation. Semin. Nephrol..

[7] Kim D.H., Kim M., Kim H., Kim Y.-L., Kang S.-W., Yang C.W., Kim N.-H., Kim Y.S., Lee J.P. (2013). Early referral to a nephrologist improved patient survival: Prospective cohort study for end-stage renal disease in Korea. PLoS ONE.

[8] Smart N.A., Dieberg G., Ladhani M., Titus T. (2014). Early referral to specialist nephrology services for preventing the progression to end-stage kidney disease. Cochrane Database Syst. Rev..

[9] Stack A.G. (2003). Impact of timing of nephrology referral and pre-ESRD care on mortality risk among new ESRD patients in the United States. Am. J. Kidney Dis..

[10] Okazaki M., Inaguma D., Imaizumi T., Kada A., Yaomura T., Tsuboi N., Maruyama S. (2018). Unfavorable effects of history of volume overload and late referral to a nephrologist on mortality in patients initiating dialysis: A multicenter prospective cohort study in Japan. BMC Nephrol..

[11] Park J., Kim M., Kim H., An J.N., Lee J., Yang S.H., Cho J.-H., Kim Y.-L., Park K.-S., Oh Y.K. (2015). Not early referral but planned dialysis improves quality of life and depression in newly diagnosed end stage renal disease patients: A prospective cohort study in Korea. PLoS ONE.

[12] Caskey F.J., Wordsworth S., Ben T., de Charro F.T., Delcroix C., Dobronravov V., van Hamersvelt H., Henderson I., Kokolina E., Khan I.H. (2003). Early referral and planned initiation of dialysis: What impact on quality of life?. Nephrol. Dial. Transplant..

[13] Walker R.C., Marshall M.R. (2014). Increasing the uptake of peritoneal dialysis in New Zealand: A national survey. J. Ren. Care.

[14] Blunt I., Bardsley M., Strippoli G.F.M. (2015). Pre-dialysis hospital use and late referrals in incident dialysis patients in England: A retrospective cohort study. Nephrol. Dial. Transplant..

[15] Stack A.G. (2002). Determinants of modality selection among incident US dialysis patients: Results from a national study. J. Am. Soc. Nephrol..

[16] Baer G., Lameire N., van Biesen W. (2010). Late referral of patients with end-stage renal disease: An in-depth review and suggestions for further actions. NDT Plus.

[17] Ethier I., Cho Y., Hawley C., Pascoe E.M., Roberts M.A., Semple D., Nadeau-Fredette A.-C., Sypek M.P., Viecelli A., Campbell S. (2020). Effect of patient- and center-level characteristics on uptake of home dialysis in Australia and New Zealand: A multicenter registry analysis. Nephrol. Dial. Transplant..

[18] Marrón B., Ostrowski J., Török M., Timofte D., Orosz A., Kosicki A., Całka A., Moro D., Kosa D., Redl J. (2016). Type of Referral, Dialysis Start and Choice of Renal Replacement Therapy Modality in an International Integrated Care Setting. PLoS ONE.

[19] MNC Medical Netcare GmbH (2017). Annual Report about the Quality in Dialysis 2016 [Jahresbericht 2016 zur Qualität in der Dialyse].

[20] Choy A.S.-M., Li P.K.-T. (2015). Sustainability of the Peritoneal Dialysis-First Policy in Hong Kong. Blood Purif..

[21] Tennankore K.K., Hingwala J., Watson D., Bargman J.M., Chan C.T. (2013). Attitudes and perceptions of nephrology nurses towards dialysis modality selection: A survey study. BMC Nephrol..

[22] Bouvier N., Durand P.-Y., Testa A., Albert C., Planquois V., Ryckelynck J.-P., Lobbedez T. (2009). Regional discrepancies in peritoneal dialysis utilization in France: The role of the nephrologist’s opinion about peritoneal dialysis. Nephrol. Dial. Transplant..

[23] Jain A.K., Blake P., Cordy P., Garg A.X. (2012). Global trends in rates of peritoneal dialysis. J. Am. Soc. Nephrol..

[24] National Association of Statutory Health Insurance Physicians (2017). Doctor’s Fee Scale: 3rd Quarter 2017. [Einheitlicher Bewertungsmaßstab (EBM): Stand: 3. Quartal 2017].

[25] National Association of Statutory Health Insurance Funds Statutory Health Insurance. https://www.gkv-spitzenverband.de/english/statutory_health_insurance/statutory_health_insurance.jsp.

[26] Federal Ministry of Health Obligation to Contract. [Kontrahierungszwang]. https://www.bundesgesundheitsministerium.de/service/begriffe-von-a-z/k/kontrahierungszwang.html.

[27] Lonnemann G., Duttlinger J., Hohmann D., Hickstein L., Reichel H. (2017). Timely Referral to Outpatient Nephrology Care Slows Progression and Reduces Treatment Costs of Chronic Kidney Diseases. Kidney Int. Rep..

[28] Gandjour A., Armsen W., Wehmeyer W., Multmeier J., Tschulena U. (2020). Costs of patients with chronic kidney disease in Germany. PLoS ONE.

[29] Hoffmann F., Haastert B., Koch M., Giani G., Glaeske G., Icks A. (2011). The effect of diabetes on incidence and mortality in end-stage renal disease in Germany. Nephrol. Dial. Transplant..

[30] Callhoff J., Jacobs H., Albrecht K., Saam J., Zink A., Hoffmann F. (2020). Factors Associated with Survey Non-Response in a Cross-Sectional Survey of Persons with an Axial Spondyloarthritis or Osteoarthritis Claims Diagnosis. Int. J. Environ. Res. Public Health.

[31] Safieddine B., Sperlich S., Epping J., Lange K., Geyer S. (2021). Development of comorbidities in type 2 diabetes between 2005 and 2017 using German claims data. Sci. Rep..

[32] Scholten N., Ohnhaeuser T., Schellartz I., von Gersdorff G., Hellmich M., Karbach U., Pfaff H., Samel C., Stock S., Rascher K. (2019). Multidimensional analysis of factors responsible for the low prevalence of ambulatory peritoneal dialysis in Germany (MAU-PD): A cross-sectional Mixed-Methods Study Protocol. BMJ Open.

[33] The German Hospital Federation Procedures. [Prozeduren (OPS)]. https://www.dkgev.de/themen/medizin-wissenschaft/medizinische-klassifikationen/prozeduren-ops/.

[34] Charlson M.E., Pompei P., Ales K.L., MacKenzie C.R. (1987). (A new method of classifying prognostic comorbidity in longitudinal studies: Development and validation. J. Chronic Dis..

[35] StataCorp (2019). Stata Statistical Software: Release 16.

[36] Wang D., Calabro-Kailukaitis N., Mowafy M., Kerns E.S., Suvarnasuddhi K., Licht J., Ahn S.H., Hu S.L. (2019). Urgent-start peritoneal dialysis results in fewer procedures than hemodialysis. Clin. Kidney J..

[37] Ivarsen P., Povlsen J.V. (2014). Can peritoneal dialysis be applied for unplanned initiation of chronic dialysis?. Nephrol. Dial. Transplant..

[38] Wojtaszek E., Grzejszczak A., Grygiel K., Małyszko J., Matuszkiewicz-Rowińska J. (2018). Urgent-Start Peritoneal Dialysis as a Bridge to Definitive Chronic Renal Replacement Therapy: Short- and Long-Term Outcomes. Front. Physiol..

[39] Li W.-Y., Wang Y.-C., Hwang S.-J., Lin S.-H., Wu K.-D., Chen Y.-M. (2017). Comparison of outcomes between emergent-start and planned-start peritoneal dialysis in incident ESRD patients: A prospective observational study. BMC Nephrol..

[40] DAK About Us. [Über uns]. https://www.dak.de/dak/unternehmen/ueber-uns-2091798.html#/.

[41] SBK Profile. [Profil]. https://www.sbk.org/unternehmen-sbk/profil/.

