# Supplementary material for: Early Referral to Nephrological Care and the Uptake of Peritoneal Dialysis. An Analysis of German Claims Data
Source: Int J Environ Res Public Health. 2021 Aug 7;18(16):8359. doi: 10.3390/ijerph18168359 (PMC8393988; doi:10.3390/ijerph18168359)
Supplement: Supplementary file 1 [file ijerph-18-08359-s001.zip › ijerph-1306242-supplementary.pdf]

Supplementary Material

Table S1. Dialysis-associated EBM and OPS codes and their operationalization for the analysis.

	Service	Operationalization for analysis
	EBM codes	
40823	In-center HD ≥ 18 years	Identification of the first HD
40824	In-center HD ≥ 18 years, < 3x per week	Identification of the first HD
40825	PD, HHD or IPD ≥ 18 years	Identification of the first PD, HHD, or IPD
40826	PD, HHD, IPD ≥ 18 years, < 4 dialysis days per week	Identification of the first PD, HHD, or IPD
40827	IPD or HHD ≥ 18 years, < 3x per week	Identification of the first HHD or IPD
40837	Addition to 40825 for IPD	Differentiation of IPD patients from PD/HHD/IPD
40838	Addition to 40827 for IPD	Differentiation of IPD patients from PD/HHD/IPD
13600	Addition for the continuous care of a patient with CKD<br>Mandatory:<br>- GFR < 40 ml/min<br>and/or<br>- Nephrotic syndrome<br>- Information about dialysis and/or transplantation<br>Optional:<br>- Counseling of relatives<br>- Preparation of dialysis and/or transplantation	Identification of the first encounter with a nephrologist
13611	Addition for the care for PD by the physician	Verification of PD patients
	OPS codes	
8-853	Hemofiltration	Identification of the first inpatient hemodialysis
8-854	Hemodialysis	Identification of the first inpatient hemodialysis
8-855	Hemodiafiltration	Identification of the first inpatient hemodialysis
8-857	Peritoneal dialysis	Identification of the first inpatient peritoneal dialysis

EBM Einheitlicher Bewertungsmaßstab, GFR glomerular filtration rate, HD hemodialysis, HHD home hemodialysis, IPD intermittent peritoneal dialysis, OPS Operationen- und Prozedurenschlüssel, PD peritoneal dialysis.
